# Emotionale Kompetenzen bei Menschen mit chronifizierten Schmerzen: Ein Selbst- und Fremdbild

**DOI:** 10.1007/s00482-023-00720-x

**Published:** 2023-06-06

**Authors:** Anne Juliane Körner, Rainer Sabatowski, Lisa Burdic, Linn Beyer, Anne Gärtner, Benjamin Schönbach, Ulrike Kaiser

**Affiliations:** 1 Universitätsklinikum Carl Gustav Carus Dresden, UniversitätsSchmerzCentrum (USC), Fetscherstr. 74, 01307 Dresden, Deutschland; 2 Klinik für Anästhesiologie und Intensivmedizin, Universitätsklinikum Schleswig-Holstein, Ratzeburger Allee 160, 23538 Lübeck, Deutschland

**Keywords:** Emotionale Expressivität, Multimodale Therapie/chronischer Schmerz, Interdisziplinäre Therapie, Selbstbeurteilung/emotionale Kompetenz, Fremdbeurteilung/emotionale Kompetenz, Emotional expressivity, Multimodal therapy/chronic pain, Interdisciplinary treatment, Self-assessment/emotional competence, External assessment/emotional competence

## Abstract

**Fragestellung:**

Ziel der Studie ist es, den Status quo der emotionalen Kompetenz (EK) von Menschen mit chronifizierten Schmerzen zu erfassen. Wie erleben sich Patient*innen selbst hinsichtlich ihrer Fähigkeiten, Emotionen wahrzunehmen, auszudrücken und zu regulieren? Und deckt sich diese Einschätzung mit der Beurteilung der EK durch psychologisches Fachpersonal?

**Methoden:**

Die Studie fand im Rahmen einer tagesklinischen interdisziplinären multimodalen Schmerztherapie an *N* = 184 erwachsenen deutschsprachigen Personen mit nichttumorbedingten, chronifizierten Schmerzen statt. EK wurde zum Therapieende mittels der Selbst- und Fremdbeurteilungsskalen (SB/FB) des Emotionale-Kompetenz-Fragebogens ermittelt. Die Fremdbeurteilung erfolgte durch das psychologische Team. Mithilfe der für den Fragebogen zur Verfügung gestellten Normstichprobe wurden Standardwerte erstellt. Diese wurden deskriptiv und inferenzstatistisch ausgewertet.

**Ergebnisse:**

Die EK wurde von den Patient*innen selbst als durchschnittlich wahrgenommen (*M*_*SB*_____*Gesamt*_ = 99,31; *SD* = 7,78). Die Psycholog*innen schätzten die EK der Patient*innen überwiegend statistisch signifikant niedriger ein (*M*_*FB*_____*Gesamt*_ = 94,70; *SD* = 7,81; *F*(1,179) = 35,73; *p* *<* 0,001; *η*^2^ = 0,17). Die emotionale Expressivität, als eine Komponente der EK, wurde als unterdurchschnittlich fremdbeurteilt (*M*_*FB_Expressivität*_ *=* 89,14; *SD* = 10,33).

**Schlussfolgerung:**

Die Patient*innen mit chronifizierten Schmerzen bewerten sich selbst als nicht eingeschränkt hinsichtlich ihrer alltäglichen Fähigkeiten zur emotionalen Wahrnehmung, Expression und Regulation. Gleichzeitig schätzt das psychologische Fachpersonal dieselben Menschen als deutlich weniger emotional kompetent ein. Offen bleibt die Frage, inwiefern die divergierenden Einschätzungen mit Beurteilungsverzerrungen erklärt werden können.

## Abstract (en)

**Research question:**

The aim of the study is to assess the status quo of emotional competence in people with chronic pain. How do patients experience themselves regarding to their ability to perceive, express, and regulate emotions? And does this assessment coincide with the assessment of emotional competence (EC) by mental health professionals?

**Methods:**

The study took place in the context of interdisciplinary multimodal pain therapy at an outpatient clinic in *N* = 184 adult German-speaking individuals with non-cancer-related chronic pain. EC was assessed at the end of therapy using the self- and third-party assessment scales of the Emotional Competence Questionnaire. The external assessment was performed by the mental health team. Standard scores were created using the norm sample provided by questionnaires. These were analyzed descriptively and inferentially.

**Results:**

Self-perceived EC was average (*M*_*self*_____*total*_ = 99.31; *SD* = 7.78). The mental health professionals predominantly rated the emotional competence of the patients significantly lower (*M*_*external*___total_ = 94,70; *SD* = 7,81; *F*(1.179) = 35,73; *p* *<* 0.001; *η*^2^ = 0.17). Emotional expressivity, as a component of EC, was externally rated as below average (*M*_*expressivity_external*_ *=* 89.14, *SD* = 10.33).

**Discussion:**

Patients with chronic pain rate themselves as unimpaired in terms of their daily emotional awareness, expression, and regulation abilities. At the same time, mental health professionals rate these same individuals as significantly less emotionally competent. The question remains open as to what extent the divergent assessments can be explained by assessment bias.

„Emotionen werden in zielrelevanten Situationen ausgelöst und signalisieren, dass etwas unserer Aufmerksamkeit bedarf. Außerdem beinhalten sie eine subjektive, physiologische und Verhaltenskomponente. Typische Charakteristika sind Instabilität, Intensität, die kurze Dauer und Gerichtetheit“ [1]. Obwohl jeder Mensch Emotionen erlebt, variiert interpersonell das Ausmaß, in dem Menschen in der Lage sind, Emotionen zu identifizieren, zu verstehen, auszudrücken, diese zu regulieren und die eigenen Emotionen bzw. die der anderen aktiv zu nutzen [2]. In der Literatur finden sich für die unterschiedlichen Fertigkeiten im Umgang mit Emotionen die zum Teil synonym verwendeten Begriffe *emotionale Kompetenz* (EK) und *emotionale Intelligenz* (EI; [3]). EK umfasst die Fertigkeiten zur Wahrnehmung von Emotionen bei sich selbst und bei anderen sowie zur Regulation und Expression von Emotionen [4]. Die EI zeigt eine große inhaltliche Nähe zur kognitiven Verarbeitung von Emotionen und deren Nutzbarkeit zur Verbesserung des Denkens [5].

Emotionale Kompetenz umfasst Fertigkeiten der Wahrnehmung, Regulation und Expression von Emotionen

EK stellt einen wichtigen Prädiktor für Gesundheit dar [6]. Als negativ erlebte Emotionen, z. B. Ärger, sind weiterhin mit Schmerzerleben assoziiert [7]. In Anbetracht dessen ist es erstaunlich, dass die dahinterstehenden EK bei Menschen mit chronifizierten Schmerzen bisher wenig erforscht sind. Es liegen bereits einige Hinweise auf eine reduzierte emotionale Wahrnehmung vor [8, 9]. Exemplarisch seien hierbei auch die Verbindung zwischen Emotionsregulationsschwierigkeiten und problematischem Opioidgebrauch [10] sowie erste Erfolge emotionsfokussierender Therapiemaßnahmen [11, 12] benannt. Aktuell existiert jedoch kein umfassender Überblick zu den verschiedenen Komponenten der EK bei Menschen mit lang andauernden Schmerzen und schmerztherapeutischer Behandlung. Wie ist der Status quo der EK? Wie gut gelingt es Menschen mit chronifizierten Schmerzen, Emotionen wahrzunehmen, auszudrücken und zu regulieren? Gerade vor dem Hintergrund einer möglichen Ausweitung therapeutischer Interventionen ist diese Frage von großer Wichtigkeit.

Die vorliegende Untersuchung möchte genau diesen Punkt fokussieren und bedient sich der Begrifflichkeit *emotionale Kompetenz*, da

1. davon ausgegangen wird, dass die emotionalen Fertigkeiten, entsprechend einer Kompetenz, erlernt und verändert werden können [13].
2. für diese Studie vorwiegend die *typische *Verhaltensneigung [14] und nicht die maximal möglichen Fertigkeiten der Patient*innen in emotionalen Situationen interessieren.
3. der Intelligenzbegriff für kognitiv assoziierte Fähigkeiten reserviert bleiben sollte [4].
4. im klinischen Kontext der Kompetenzbegriff gebräuchlich ist, beispielsweise im verhaltenstherapeutischen emotionalen Kompetenztraining [15].

Folgende Forschungsfragen sollen geklärt werden:

- Wie kompetent sind Menschen mit chronischen Schmerzen in schmerzspezifischer Therapie im emotionalen Erkennen, Regulieren und Ausdrücken?
- Wie stark ist der Zusammenhang zwischen Selbst- und Fremdeinschätzung der EK?

Vor dem Hintergrund bisheriger Forschung wurden folgende Hypothesen entwickelt:

- Patient*innen mit chronifizierten Schmerzen in einer tagesklinischen interdisziplinären multimodalen Schmerztherapie (IMST) weisen – im Vergleich zur Normalbevölkerung – eine verminderte EK auf.
- Der Zusammenhang zwischen Selbst- und Fremdbeurteilung der EK ist bei Personen mit chronifizierten Schmerzen gering. Es bestehen signifikante Unterschiede.

## Material und Methoden

### Stichprobe

In diese Untersuchung wurden Patient*innen eingeschlossen, die sich in der tagesklinischen IMST des UniversitätsSchmerzCentrums Dresden befanden und die Einschlusskriterien für eine IMST erfüllten, beispielsweise die wesentliche Beteiligung psychosozialer Faktoren [16]. Es handelte sich dabei um erwachsene Menschen mit chronifizierten, nichttumorbedingten Schmerzen. Die Daten wurden von Januar 2018 bis Juni 2019 erfasst. Von den zu Therapiebeginn aufgenommenen 209 Personen schlossen 184 Menschen die Therapie nach 4 Wochen ab und waren außerdem mit der Studie einverstanden (Tab. 1).

**Table 1:** 

Alter	*M* = 50,06	*SD* = 11,28	*Min.* = 18 Jahre	*Max.* = 79 Jahre
Geschlecht	Weiblich	Männlich	–	–
	72,8 %	27,2 %	–	–
Schmerzchronifizierungsstadium nach dem Mainzer Stadienmodell [17]	I	II	III	–
	4,3 %	56 %	39,7 %	–
Schmerzlokalisation	Rücken	Kopf	Sonstige	–
	52,2 %	15,2 %	32,6 %	–
Schmerzbeginn	Vor >5 Jahren	Vor 2–5 Jahren	Vor 1–2 Jahren	Vor weniger als einem Jahr
	58,7 %	25,5 %	13 %	1,6 %

### Setting

Der Behandlungszeitraum der tagesklinischen IMST umfasste insgesamt 5 Wochen. Die Behandlung setzte sich zusammen aus einer 4‑wöchigen Hauptbehandlungsphase und einer sich nach 10 Wochen Alltagserprobung anschließenden Auffrischungswoche. Vor Beginn der tagesklinischen Behandlung erfolgte ein umfangreiches multiprofessionelles Assessment [18]. Alle Eingangsbefunde wurden in den interdisziplinären Teambesprechungen diskutiert. Die Schmerztherapie selbst orientierte sich an den Behandlungselementen der *Ad-hoc-Kommission Interdisziplinäre Multimodale Schmerztherapie* der Deutschen Schmerzgesellschaft e. V. [19]. Ein emotionsfokussiertes Behandlungselement war beispielsweise die tägliche emotionsbezogene Befindensrunde in der Gruppenpsychotherapie.

### Instrument

Die *EK* wurde mittels der Selbst- und Fremdbeurteilungsskalen (SB/FB) des Emotionale-Kompetenz-Fragebogens (EKF; [4]) ermittelt. Die Formulierungen der Items sind analog konzipiert (SB: erste Person Singular; FB: dritte Person Singular). Die Bearbeitungszeit des EKF in der Selbst- und Fremdbeurteilungsvariante beträgt 10–20 min. Der Fragebogen besteht aus 4 Hauptskalen: *Erkennen eigener Emotionen, Erkennen von Emotionen bei anderen, Regulation und Kontrolle eigener Emotionen und emotionale Expressivität.* Für den Fragebogen liegen Normstichproben und Referenzwerte vor (*N*_Normstichprobe_SB_ *=* 638; *N*_Normstichprobe_FB_ *=* 421; [4]).

### Prozedere

Die Selbst- und Fremdbeurteilung fand am Ende der 4‑wöchigen Therapie statt. Die SB-Instrumente wurden der standardmäßigen Therapieevaluation beigefügt. Die FB-Fragebögen des EKF wurden von den jeweiligen psychologischen Bezugstherapeut*innen (*n*_männlich_ = 1; *n*_weiblich_ = 4; *M* = 32,2 Jahre) ausgefüllt. Die Fremdbeurteilenden verfügten über 4–7 Jahre berufspraktische Erfahrung in der IMST. Der Messzeitpunkt wurde so gewählt, dass das psychologische Personal ausreichend Gelegenheit hatte, die zu beurteilenden Personen umfassend kennenzulernen.

### Statistische Auswertung

Zunächst wurden die Rohwerte (X) durch Abgleich mit der Normstichprobe des EKF (SB: 61,5 % weiblich, *M* = 21,7 Jahre, 78 % Auszubildende; FB: 58 % weiblich, *M* = 27,3 Jahre [4]) in Standardnormwerte umgewandelt: ((X−*M*_Normstichprobe_/*SD*_Normstichprobe_) × 0,1) [4]. Pro Person konnte anhand des Standardnormwerts jeweils die Ausprägung der EK im Vergleich zur nichtklinischen Normstichprobe festgestellt werden. Dabei galt eine Ausprägung zwischen 90 und 110 als durchschnittlich, Werte kleiner 90 als unterdurchschnittlich und über 110 als überdurchschnittlich [4]. Es wurden die mittlere EK-Ausprägung der Gesamtstichprobe und deren Standardabweichung erfasst. Nach Überprüfung der statistischen Voraussetzungen fand ein inferenzstatistischer Vergleich von Selbst- und Fremdbeurteilung statt. Trotz der oft überprüften Robustheit der einfaktoriellen Varianzanalyse mit Messwiederholung gegenüber der Normalverteilungsvoraussetzung wurden die Ergebnisse der Subskalenvergleiche aufgrund nicht vollständig erfüllter Voraussetzungen mithilfe des nichtparametrischen Wilcoxon-Tests abgesichert. Zudem wurde die Spearman-Rang-Korrelation angewendet.

## Ergebnisse

### Selbstbeurteilung der emotionalen Kompetenz

Patient*innen beurteilten sich im Mittel als durchschnittlich hinsichtlich ihrer EK (Normbereich: 90–110; Tab. 2). Die Einschätzung der Patient*innen hinsichtlich ihrer Kompetenzen im *Erkennen, Regulieren* und *Ausdrücken *von Emotionen lag ebenfalls im Normbereich.

**Table 2:** 

		*M*	*SD*	*df*_*zw*_	Standardteststatistik	*p*	Effektstärke^a^
Emotionale Kompetenz (Gesamtwert)	SB	99,31	7,78	1	*F* = 35,73	*p* *<* 0,001	*η*_p_^2^ = 0,17
	FB	94,70	7,81				
Erkennen eigener Emotionen	SB	99,13	10,49	1	*z* *=* −3,19	*p* *=*0,001	*r* *=* 0,24
	FB	94,43	13,01				
Erkennen von Emotionen bei anderen	SB	100,32	11,28	1	*z* *=* −1,45	*p* *=* 0,148	*r* *=* 0,11
	FB	98,48	12,68				
Regulation eigener Emotionen	SB	100,19	9,37	1	*z* *=* −4,16	*p* *<* 0,001	*r* *=* 0,31
	FB	96,74	8,37				
Emotionale Expressivität	SB	97,53	11,03	1	*z* *=* −7,45	*p* *<* 0,001	*r* *=* 0,56
	FB	89,14	10,33				

*df*_*zw*_ Freiheitsgrade im Within-Vergleich, *FB* Fremdbeurteilung, *M* Mittelwert – Standardnormwerte geben durch Verrechnung mit dem vom Fragebogenautor zur Verfügung gestellten Mittelwert und der Standardabweichung der Normstichprobe Aufschluss über die relative Position des Rohwerts innerhalb der Normstichprobe, Interpretation der Standardnormwerte: 90–110 durchschnittliche Ausprägung, < 90 unterdurchschnittliche Ausprägung, *p* Wahrscheinlichkeitsmaß zur Feststellung der Signifikanz in Hypothesentests, *r* und *η*_p_^2^ Effektstärkemaß nach Cohen [20]: 0,01 kleiner/0,06 mittlerer/0,14 großer Effekt; *r* = 0,10 kleiner Effekt, *r* = 0,30 mittlerer Effekt, *r* = 0,5 großer Effekt, *SB* Selbstbeurteilung, *SD* Standardabweichung, Wilcoxon*-z* standardisiertes Ergebnis des nichtparametrischen Signifikanztests, falls die Voraussetzungen für eine parametrische Testung nicht vollständig erfüllt waren

^a^Die A‑posteriori-Power, einen mittelstarken Effekt zu detektieren, lag bei einer 2‑seitigen Testung (α/2 = 2,5 %) bei 100 %

### Fremdbeurteilung der emotionalen Kompetenz

Auch das psychologische Behandlungsteam der Tagesklinik beurteilte die untersuchten Personen überwiegend als durchschnittlich emotional kompetent. Die fremdeingeschätzten EK *Erkennen von Emotionen bei sich/anderen* und *Regulieren von Emotionen* lagen im Normbereich (90 bis 110; Tab. 2). Lediglich die emotionale *Expressivität* der Patient*innen wurde von den psychologischen Einschätzenden im Mittel als unterdurchschnittlich (*M*_*FB_Expressivität*_ *=* 89,14; Tab. 2) beurteilt.

### Signifikante Unterschiede zwischen Selbst- und Fremdeinschätzung

**Figure 1:**
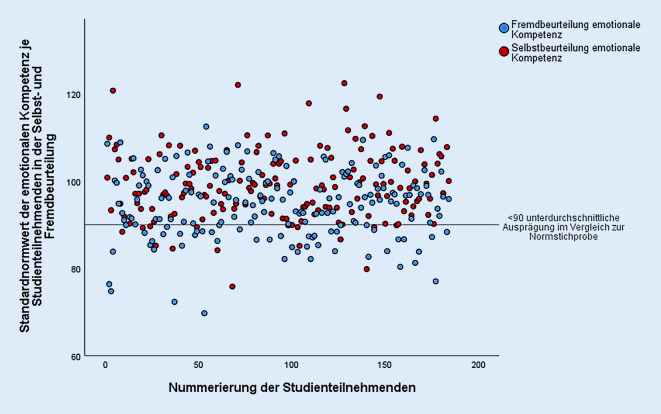

In Abb. 1 wird exemplarisch die Verteilung von Selbst- und Fremdbeurteilung der EK auf Gesamtskalenebene dargestellt.

Inferenzstatistische Überprüfungen zeigten, dass die psychologische Fremdbeurteilung im Mittel deutlich niedriger ausfiel (*M*_SB_ = 99,3, *M*_FB_ = 94,7; Tab. 2). Tatsächlich unterschied sich die fremdbeurteilte EK auf *Gesamtskalenebene* in großem Ausmaß von der Selbstbeurteilung (*F*(1,179) = 35,73; *p* *<* 0,001; *η*^2^ = 0,17; Tab. 2). Bei der detaillierten Aufschlüsselung zeigte sich der gleiche Effekt bei den Kompetenzen *Wahrnehmung von Emotionen bei sich selbst* (z = −3,19; *p* = 0,001; *r* = 0,24), *Expressivität *(*z* = −7,45; *p* < 0,001;* r* = 0,56) und *emotionale Regulation* (*z* = −4,16; *p* < 0,001; *r* = 0,31), nicht jedoch bei der EK *Wahrnehmung von Emotionen bei anderen* (z = −1,45; *p* = 0,148; *r* = 0,11; Tab. 2).

### Schwacher Zusammenhang zwischen Selbst- und Fremdeinschätzung

Bei der Analyse der Spearman-Korrelationen (Tab. 3) zeichnete sich ein signifikant geringer Zusammenhang zwischen den *Gesamtwerten* der selbst- und fremdeingeschätzten EK (*r*_s_ = 0,19; *p* *<* 0,05) ab. Selbst- und Fremdeinschätzung korrelierten hinsichtlich der *emotionalen Regulationskompetenz* (*r*_s_ = 0,24; *p* < 0,001) und *Expressivität* (*r*_s_ = 0,28; *p* < 0,001) gering. Hinsichtlich des *Erkennens von Emotionen bei anderen* gab es einen mittleren Zusammenhang (*r*_s_ = 0,31; *p* < 0,001). Die Selbst- und Fremdeinschätzung der Fähigkeit zum *Erkennen von Emotionen bei sich selbst* korrelierten nicht miteinander (*r*_s_ = 0,05).

**Table 3:** 

		FB (T2)				
		Emotionale Kompetenz (Gesamt)	Erkennen eigener Emotionen	Erkennen von Emotionen bei anderen	Emotionale Regulation	Emotionale Expressivität
SB (T2)	Emotionale Kompetenz (Gesamt)	0,19^a^	0,12	0,11	0,22^a^	0,19^a^
	Erkennen eigener Emotionen	0,03	0,05	−0,124	0,23^a^	0,03
	Erkennen von Emotionen bei anderen	0,21^a^	0,13	0,31^b^	0,02	0,15^a^
	Emotionale Regulation	0,03	−0,03	0,02	0,24^b^	−0,02
	Emotionale Expressivität	0,18^a^	0,13	0,03	0,11	0,28^b^

*FB* Fremdbeurteilung, *SB* Selbstbeurteilung, *T2* Therapieende

Interpretation von *r*_s_ nach Cohen [20]: |*r*_s_| = 0,10 schwache, |*r*_s_| = 0,30 mittlere/moderate, |*r*_s_| = 0,50 große/starke Korrelation

^a^Signifikante Korrelation bei einseitiger Testung und einem Signifikanzniveau von *α* = 5 %/*p* *<* 0,05

^b^Signifikante Korrelation bei einseitiger Testung und einem Signifikanzniveau von *α* = 0,1 %/*p* *<* 0,001

## Diskussion

Die Patient*innen bewerteten sich selbst als *nicht eingeschränkt* im emotionalen Wahrnehmen, Regulieren und Ausdrücken. Auch die Einschätzungen des gesamten psychologischen Teams zur EK der Patient*innen lagen überwiegend im Normbereich. Einzige Ausnahme bildete die als deutlich vermindert beurteilte emotionale Expressivität. Letzteres legt nahe, dass Personen mit chronifizierten Schmerzen auf andere den Eindruck vermittelten, Emotionen nonverbal oder verbal wenig mitzuteilen. Die reduziert wahrgenommene emotionale *Expressivität* geht einher mit der in der Literatur auffindbaren Evidenz zum Einfluss der Unterdrückung von Ärger auf das Schmerzerleben [21, 22]. Diskrepanzen bestehen jedoch zwischen den durchschnittlichen selbsteingeschätzten Kompetenzen des *emotionalen Erkennens* und *Regulierens* und anderen Forschungsergebnissen. So konnten in einer Übersichtsarbeit [23] zur *emotionalen Regulation *bei chronischem Schmerz bei einem Teil der Untersuchungen direkte Zusammenhänge zwischen maladaptiven emotionalen Regulationsfähigkeiten und erhöhtem Schmerzerleben nachgewiesen werden. Bisherige Forschung mit schmerzhomogenen und -heterogenen Stichproben extrahierte weiterhin reduzierte Fertigkeiten im *emotionalen Erkennen* [8, 24, 25]. Von Korn et al. [9] konnten im Gesichtserkennungstest bei Patient*innen einer Rehabilitationsklinik mit chronischen Kreuzschmerzen einerseits zeigen, dass sie Emotionen genauso kompetent erkannten wie die Normalbevölkerung. Andererseits differenzierten sie, dass die untersuchten Personen mit Schmerzchronifizierungsgrad 3 und 4 auf der Graded Chronic Pain Scale (28,5 %) sich selbst als eingeschränkt im Erkennen von Emotionen beschrieben. Daraus ableitend stellt sich die Frage, ob sich lediglich eine Subgruppe der Patient*innen mit chronischen Schmerzen als (reduziert) kompetent erlebt und inwiefern das Chronifizierungsstadium dabei eine Rolle spielt. Dieser Aspekt sollte Gegenstand weiterführender Untersuchungen sein.

In der vorliegenden Studie bestand ein schwacher Zusammenhang zwischen Selbst- und Fremdeinschätzung

Im Gegensatz zur vorliegenden Studie, in der ein schwacher Zusammenhang zwischen Selbst- und Fremdeinschätzung bestand, korrelierten Selbst- und Fremdbeurteilung in der Normstichprobe des EKF mittelstark [4]. In Diskrepanz zum Manual, in dem die Allgemeinbevölkerung befragt wurde, lag in dieser Studie a priori eine höhere Homogenität der Beurteilungsgruppen vor: In der Selbstbeurteilung waren nur Menschen mit chronifizierten Schmerzen und in der Fremdbeurteilung ausschließlich Psycholog*innen involviert. Der klinische Kontext und die damit verbundene Stichprobenauswahl werden als vorrangige Erklärung für die Unterschiede eingestuft. Außerdem war die Normstichprobe im Durchschnitt jünger (Differenz SB: −38,4; FB: −4,9 Jahre). In Anbetracht dessen, dass sich der Umgang mit Emotionen im Laufe des Lebens zu verbessern scheint [4], können an dieser Stelle Alterseffekte nicht ausgeschlossen werden.

Tatsächlich unterschieden sich die Einschätzungen von Patient*innen und Psycholog*innen innerhalb des Normbereichs. Das psychologische Behandlungsteam beurteilte die Patient*innen innerhalb des Normbereichs als weniger kompetent, als die untersuchten Personen sich selbst beurteilten. Lediglich bei der Einschätzung zur *Wahrnehmung von Emotionen bei anderen* gab es keine signifikanten Unterschiede. Die ansonsten fehlende Kongruenz von Selbst- und Fremdeinschätzung ist bei Menschen mit chronifizierten Schmerzen auch an anderer Stelle bekannt: So beurteilen die Behandelnden die Schmerzstärke [26] deutlich geringer als die Patient*innen selbst. Inwiefern ein positiver oder negativer Zusammenhang zwischen der emotionalen Expressivität und dem für andere sichtbaren Ausdruck von Schmerz besteht, könnte für die weitere Forschung interessant sein.

In der vorliegenden Untersuchung haben mit hoher Wahrscheinlichkeit allgemeingültige sozialpsychologische Beobachtungs- und Erinnerungseffekte Einfluss genommen (beispielsweise FB: *Korrespondenzverzerrung* [27], *„first impression“ *[28], *stereotype Vorannahmen*; SB: *„recall bias“ *[29], sozial erwünschtes Antworten). Offen ist, in welchem Ausmaß diese Verzerrungen eine Rolle spielten.

Unabhängig von diesen Beurteilungseffekten sind die Ergebnisse weiterhin nur auf Patient*innen in einer tagesklinischen IMST übertragbar. Es handelt sich um eine selektive Gruppe mit einem hohen Maß an Beeinträchtigungen und gehäuft erfolglosen Vorbehandlungen [16, 19]. Ob die Evidenzen ebenso für Personen mit chronischen Schmerzen ohne IMST zutreffen, ließ sich mit dem in dieser Studie gewählten Design nicht eruieren. Die Ergebnisse sind zudem nur auf Menschen mit einem deutschen kulturellen Hintergrund übertragbar.

In der Konzeptualisierung der Studie und der zugehörigen Recherchearbeit war die häufig fehlende Trennschärfe der Konzepte *EI* und *EK* ein erschwerender Faktor. Ein Beispiel hierfür ist die unzureichende Unterscheidung in maximale (EI im „klassischen Sinne“) vs. im Alltag typischerweise gezeigte emotionale Fertigkeiten (EK). Letztlich können die Ergebnisse dieser Untersuchung zur EK nur dahingehend betrachtet und generalisiert werden, wie EK für diese Studie als Konstrukt definiert wurde (siehe Einleitungsteil).

## Ausblick

Von Interesse für die weitere Forschung sollte es sein, die unterschiedliche Selbst- und Fremdbeurteilung der EK besser zu verstehen. Durch ein auf Beobachtungs- und Selbstbeurteilungseffekte ausgerichtetes Studiendesign sollte der gefundene Unterschied genauer überprüft und kontrolliert werden. Es ist zudem sinnvoll, die Fremdbeurteilungen anderer Fachdisziplinen oder der Angehörigen vergleichend einzubeziehen.

Weiterhin empfiehlt sich, die Erfassung der EK noch stärker zu objektivieren. Einen Ansatzpunkt hierfür bietet die Untersuchung emotionaler Fertigkeiten mithilfe zusätzlicher physiologischer Parameter wie Herzratenvariabilität, Hautleitfähigkeit, Atemfrequenz und muskulärer Anspannung. Sicherlich braucht es hierfür zunächst die Definition dessen, was EK auf dieser Ebene bedeutet. Genauere physiologische Marker für die Fertigkeiten des emotionalen Erkennens, Regulierens und Ausdrückens sollten hierfür bestimmt werden. Mögliche Verzerrungen der Selbst- und Fremdbeurteilungen würden sich bei der Erfassung durch physiologische Parameter erübrigen.

Die Ergebnisse dieser Studie bieten einen Orientierungspunkt für die schmerztherapeutische Gesprächsführung und Behandlung. Mit dem Wissen um die klinisch relevanten Unterschiede in der wahrgenommenen emotionalen Expressivität empfiehlt sich für Behandelnde ein sensibles Explorieren, Verbalisieren und Verstärken gezeigter Emotionen. Vor dem Hintergrund erster Evidenzen zum inkrementellen Wert einer gezielten Förderung emotionaler Expressivität in der Schmerztherapie [11, 12] und angesichts der in dieser Untersuchung gefundenen Hinweise zum Status quo lohnt sich für die zukünftige Forschung und Behandlung der weiterführende Blick in Richtung eines emotional kompetenten Ausdrucks von Emotionen.

## Fazit für die Praxis

- Emotionale Kompetenz (EK) umfasst die Fertigkeiten zur Wahrnehmung von Emotionen bei sich selbst und bei anderen sowie zur Regulation und Expression von Emotionen.
- Die EK ist ein wichtiger Prädiktor für Gesundheit. Bei Menschen mit chronifizierten Schmerzen ist sie bisher wenig umfassend erforscht.
- Die Patient*innen mit tagesklinischer interdisziplinärer multimodaler Schmerztherapie bewerteten sich in der vorliegenden Studie selbst als nicht eingeschränkt im emotionalen Wahrnehmen, Regulieren und Ausdrücken. Auch die Einschätzungen des psychologischen Teams lagen überwiegend im Normbereich. Hingegen wurde die emotionale Expressivität von den Psycholog*innen als deutlich vermindert beurteilt.
- Das psychologische Behandlungsteam beurteilte die Patient*innen innerhalb des Normbereichs meist als weniger kompetent. Die untersuchten Personen schätzten sich selbst vergleichsweise emotional kompetenter ein.
- Für zukünftige Forschung und Behandlung lohnt sich der weiterführende Blick in Richtung einer emotional kompetenten Expression von Emotionen bei Menschen mit chronifizierten Schmerzen.
